# Lung sparing and ribcage coverage in total body irradiation delivered by helical tomotherapy

**DOI:** 10.1186/s40001-022-00918-2

**Published:** 2022-12-10

**Authors:** Mümtaz Köksal, Jonathan Baumert, Felix Schoroth, Davide Scafa, David Koch, Christina Leitzen, Gustavo R. Sarria, Frank A. Giordano, Georgios Chatzikonstantinou, Leonard C. Schmeel

**Affiliations:** 1 Department of Radiation Oncology, University Hospital Bonn, Bonn, Germany; 2 Department of Radiation Oncology, University Medical Centre Mannheim, Mannheim, Germany; 3 Department of Radiation Oncology, University Hospital Frankfurt, Frankfurt, Germany

**Keywords:** Lungs, Total body irradiation, Helical tomotherapy, Dose sparing, Bone-marrow transplantation

## Abstract

**Purpose:**

Helical tomotherapy (HT) is a viable method for delivering total body irradiation (TBI) when preparing patients for allogenic stem cell or bone-marrow transplantation. TBI can be planned to reduce the amount of radiation delivered to organs at risk, such as the lungs, with the aim of decreasing toxicity. However, it is important for the ribcage to receive the prescribed radiation dose in preparation for bone-marrow transplantation. In this retrospective study, we analyzed radiation dose coverage of the lungs and ribcage in patients who underwent TBI delivered by HT to achieve lung dose sparing.

**Methods:**

Thirty-five patients were included in the analysis and divided into three groups based on their prescribed radiation dose (4, 8, or 12 Gy). HT was performed using a rotating gantry to reduce radiation to the lungs. Dosimetric parameters for the lungs and ribcage as well as dose-volume histograms were calculated.

**Results:**

The mean lung D_95_ was 60.97%, 54.77%, and 37.44% of the prescribed dose for patients receiving 4 Gy, 8 Gy, and 12 Gy, respectively. Ribcage coverage was most optimal for patients receiving 4 Gy, with a D_95_ of 91.27% and mean homogeneity index of 1.17, whereas patients receiving 12 Gy had a mean D_95_ of 78.65% and homogeneity index of 1.37, which is still within the range recommended by treatment guidelines.

**Conclusions:**

Using HT to achieve lung tissue sparing is a viable approach to minimizing pulmonic complications in patients undergoing TBI. As this planning adjustment does not compromise the dose and quality of coverage received by the ribcage, it is a feasible tool within conditioning regimens for allogeneic bone-marrow transplantation.

## Introduction

Alongside chemotherapy, total body irradiation (TBI) is a major component of the conditioning regimen in preparation for allogenic bone-marrow transplantation (BMT) in patients with diseases, such as acute lymphatic leukemia (ALL) or acute myeloid leukemia (AML) [1, 2]. TBI helps eradicate radiosensitive malignant cells and is immunosuppressive, which increases the likelihood of donor transplant acceptance by the patient’s body. However, the toxicity of TBI can limit its use in conditioning regimens, especially considering organs at risk (OAR), such as the lungs [3], which can be vulnerable to complications including interstitial pneumonia [4]. To reduce the risk of such side effects, approaches such as using lung blocks to reduce the amount of radiation reaching the lungs can be employed [5]. However, lung blocks can also severely reduce the dose of radiation delivered to the mediastinum and ribs, which is important in the preparation for allogenic BMT.

Our institution delivers TBI using helical tomotherapy (HT), which minimizes differences between planned and delivered doses and increases the overall homogeneity of irradiation across the body [6–8]. Lung sparing in TBI reduces the risk of pulmonic side effects and long-term problems resulting from radiation-induced lung damage [9]. However, when using HT to reduce the dose delivered to the lungs, it is of utmost importance to prohibit excessive reductions in the dose delivered to the ribcage, which can lead to an increased risk of relapse of the original disease [10]. A previous simulation study suggests that HT may be a feasible method of delivering the prescribed dose to the ribcage and planning target volume (PTV) while reducing lung irradiation [11]. Here, we analyzed data from 35 patients at our institution who underwent TBI using HT as part of their bone-marrow conditioning regimen. We demonstrate the feasibility of using HT to reduce irradiation of lung tissue, thereby minimizing risks of side effects or long-term health issues while maintaining the radiation dose prescribed to the ribcage to lessen the likelihood of relapse.

## Materials and methods

As our data were obtained for routine quality assurance, which is standard of care in our institution, and are in line with requirements of the German radiation protection law, ethical approval was not required.

### Patients

We retrospectively analyzed data from all patients undergoing TBI in our institution between 2012 and 2020 whose treatment plan and delivery involved sparing of lung tissue.

### Treatment planning

Two computed tomography (CT) scans were performed for each patient to delineate and plan for TBI using TomoTherapy^®^ Hi-ART II. Because TomoTherapy^®^ Hi-ART II has a maximum couch shift of 135 cm, table rotation was needed between upper body and lower body CT scans, thus requiring two treatment plans that were merged following further technical considerations [12]. As the junction between scans is located at the thighs, only data from the upper body scan were analyzed in this study. CT scans were acquired in cranio-caudal alignment with a slice thickness of 5 mm.

**Fig. 1:**
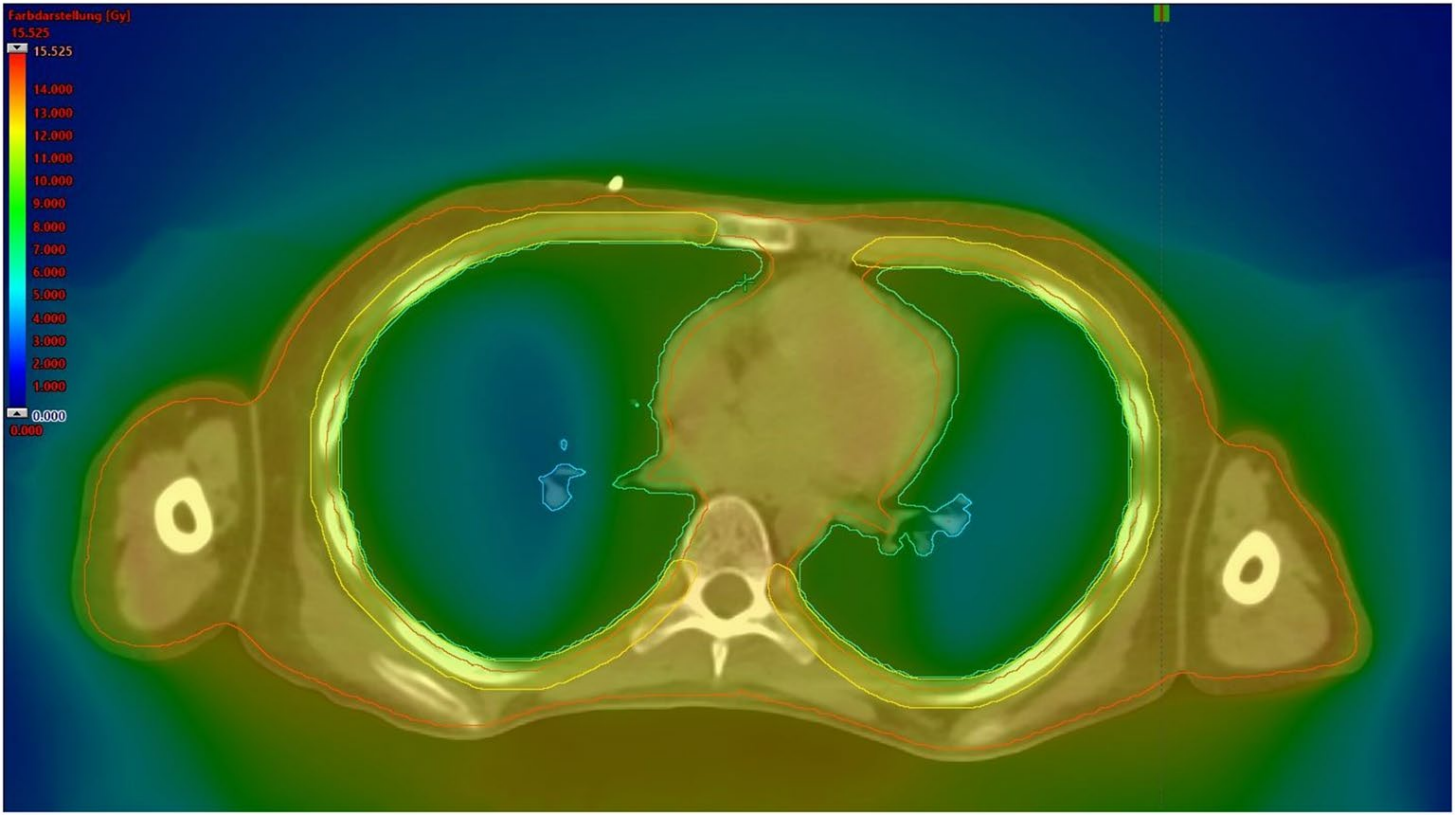
Example radiation plan with contouring of the lungs (green), ribcage (yellow), and PTV (red)

An Eclipse treatment planning system (Varian Medical System, Palo Alto, CA) was used for all delineations and planning to optimize preparation for treatment. The contouring of OARs was performed according to current institutional and international standards [13]. The PTV was also contoured. Special care was taken to accurately contour the lungs and ribcage so as to evaluate dose sparing to the lungs as well as the homogeneity and correct delivery of radiation to the ribcage according to a previously described method used at our institution [12]. The lungs were delineated using a standardized template that covers internal lung tissue up to 1 cm below the lung surface, and ribcage contouring was individually adjusted for each patient (Fig. 1). A 2.5 cm field width, pitch of 0.390, and planned modulation factor of 2.7 were used as setup planning parameters.

During TBI, patient position was fixed using a vacuum cushion for the body and fixation mask for the head to reduce movement, which could alter delivery of the planned irradiation. The beam-on time was 32.42 min on average (standard deviation (SD), 6.5 min) to deliver HT to the whole body. However, as additional time was needed for preparation and further considerations, each patient was given a 90 min timeslot.

We analyzed the efficiency of radiation dose reduction to the lungs and the resultant radiation dose delivered the ribcage during TBI. Patients were divided into three groups by their prescribed dose (4 Gy (2 × 2 Gy), 8 Gy (4 × 2 Gy), or 12 Gy (6 × 2 Gy), because the percentile dose reduction varies among these doses due to overall lower toxicity of lung tissue when irradiated with 4 Gy compared with 12 Gy [14, 15]. We also generated dose volume histograms (DVHs) and calculated the homogeneity index (HI) to examine ribcage dose exposure relative to the achieved lung dose reduction in each group. HI was calculated using the formula proposed by Kataria et al. (HI = D_5_/D_95_) [16]. Statistical analysis was performed using SPSS v26.0 (IBM, Armonk, New York, USA).

## Results

We analyzed data from 35 patients (18 women and 17 men) with an average age of 40.2 years (SD, 15.9; range, 13–72). Patients were being treated for AML (*n* = 9), ALL (*n* = 21), mixed phenotype acute leukemia (*n* = 2), mast cell leukemia (*n* = 2), diffuse large B-cell lymphoma (*n* = 1), or anaplastic large cell lymphoma (*n* = 1). Fifteen patients were planned to receive 12 Gy, 15 to receive 8 Gy, and four to receive 4 Gy.

**Fig. 2:**
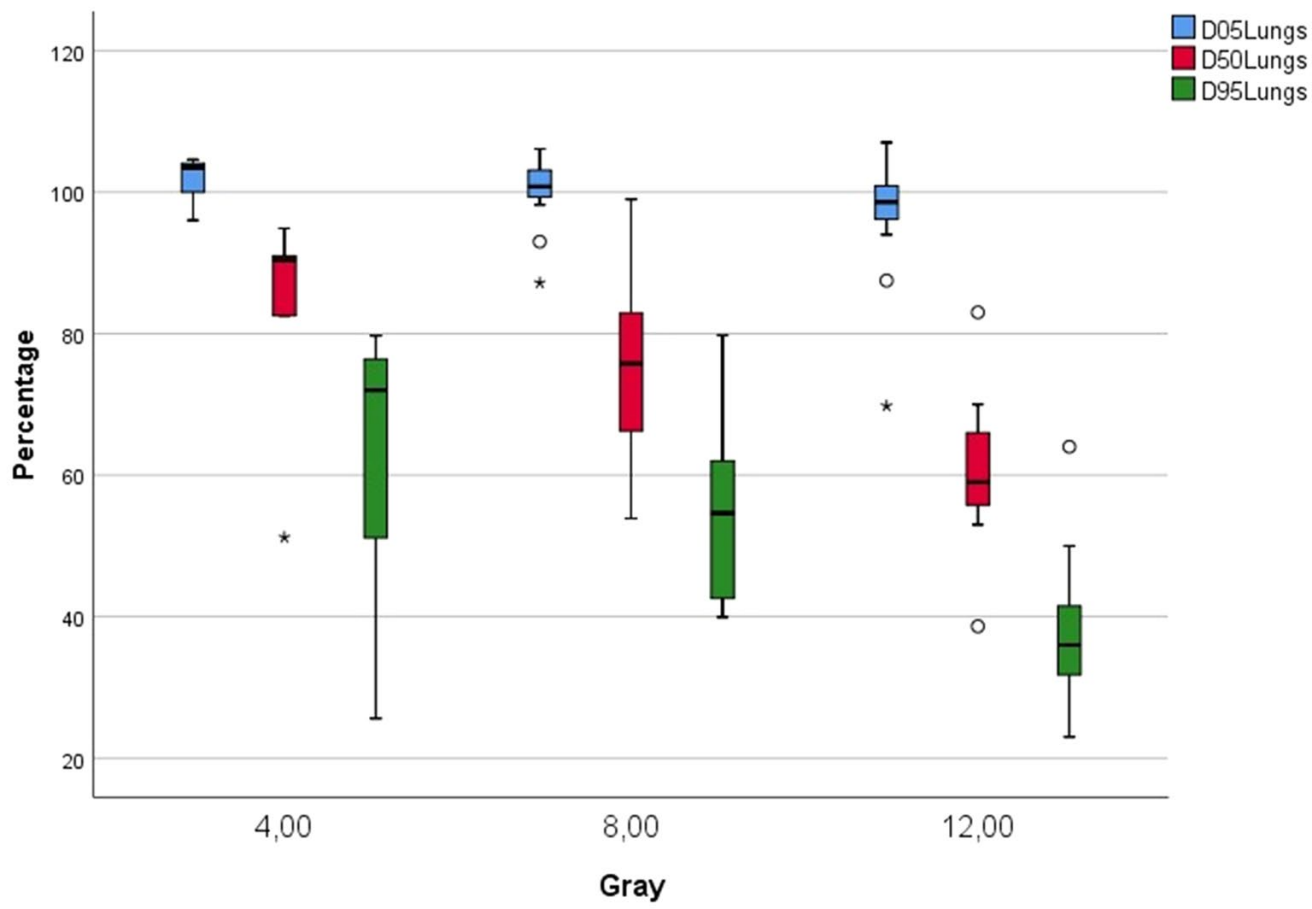
Lung dose sparing for patients in the 4 Gy, 8 Gy, and 12 Gy prescribed dose groups

Considering the lungs, patients in the 4 Gy group had a D_95_ of 60.97 ± 22.68% (mean ± SD) and D_5_ of 101.63 ± 3.62% relative to the prescribed dose (Table 1, Fig. 2), resulting in actual doses of 2.44 Gy over 95% of the lung volume and 4.07 Gy over 5% of the lung volume, respectively. Patients in the 8 Gy and 12 Gy groups had a D_95_ of 54.77 ± 13.20% and 37.44 ± 10.28% relative to the prescribed dose, resulting in doses of 4.38 Gy and 4.49 Gy over 95% of the lung volume, respectively.

**Table 1:** Observed radiation dose over the lungs relative to the prescribed dose

Planned dose	*N*	Minimum (%)	Maximum (%)	Mean (%)	SD (%)
4 Gy					
D_95_	5	25.60	79.69	60.97	22.68
D_50_	5	51.21	94.86	82.00	17.79
D_5_	5	96.01	104.60	101.63	3.62
8 Gy					
D_95_	15	39.95	79.76	54.77	13.20
D_50_	15	53.89	99.00	75.28	13.85
D_5_	15	87.17	106.11	100.15	4.78
12 Gy					
D_95_	15	23.00	64.00	37.44	10.28
D_50_	15	38.63	83.00	60.39	9.87
D_5_	15	69.78	107.00	96.68	8.67

Considering the ribcage, patients in the 4 Gy group had a D_95_ of 91.27 ± 6.16% relative to the prescribed dose, whereas patients in the 8 Gy and 12 Gy groups had a D_95_ of 89.14 ± 9.32% and 78.65 ± 11.59%, respectively (Table 2). However, patients in the 4 Gy, 8 Gy, and 12 Gy groups had similar a D_5_ of 106.01 ± 2.13%, 105.60 ± 2.10%, and 105.82 ± 2.75%, respectively.

**Table 2:** Observed radiation dose over the ribcage relative to the prescribed dose

Planned dose	*N*	Minimum (%)	Maximum (%)	Mean (%)	SD (%)
4 Gy					
D_95_	5	81.93	98.81	91.27	6.16
D_50_	5	97.00	102.76	100.42	2.16
D_5_	5	103.00	108.03	106.031	2.13
8 Gy					
D_95_	15	71.04	100.93	89.14	9.32
D_50_	15	90.42	106.11	99.81	4.21
D_5_	15	102.04	110.00	105.60	2.10
12 Gy					
D_95_	15	57.32	95.06	78.65	11.59
D_50_	15	78.50	105.00	96.43	6.34
D_5_	15	100.93	114.00	105.82	2.75

The HI ranged from 1.17 ± 0.09 in the 4 Gy group to 1.37 ± 0.20 in the 12 Gy group (Table 3).

**Table 3:** Homogeneity index

Planned dose	*N*	Minimum	Maximum	Mean	SD
4 Gy	5	1.09	1.31	1.17	0.09
8 Gy	15	1.06	1.44	1.20	0.12
12 Gy	15	1.11	1.76	1.37	0.20

**Fig. 3:**
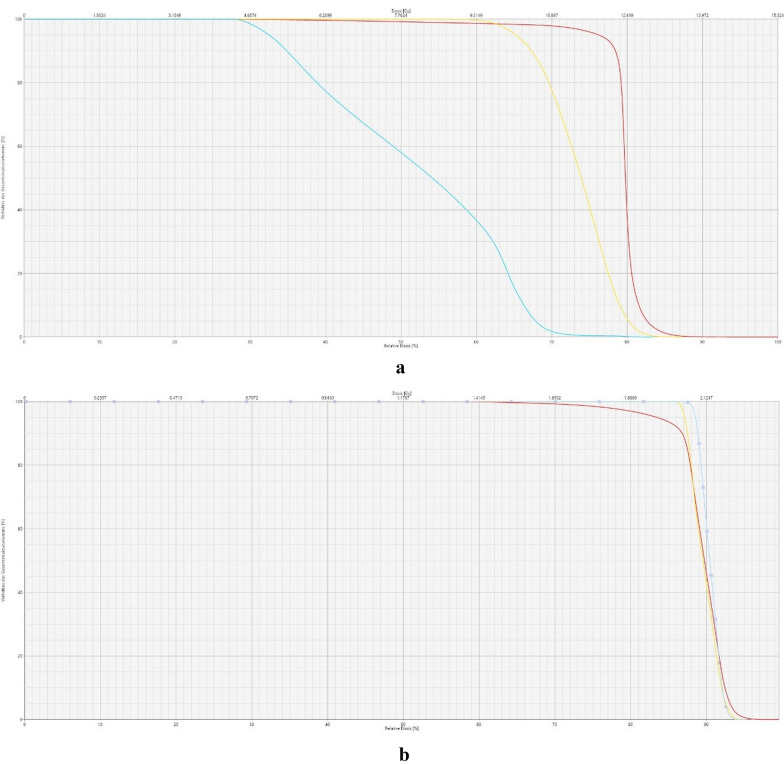
**a** DVH for lung (blue), ribcage (yellow), PTV (red) showing an example of a plan with a prescription dose of 12 Gy and lung dose sparing. **b** DVH for lung (blue), ribcage (yellow), PTV (red) showing an example of a plan with a prescription dose of 12 Gy without lung dose sparing

**Fig. 4:**
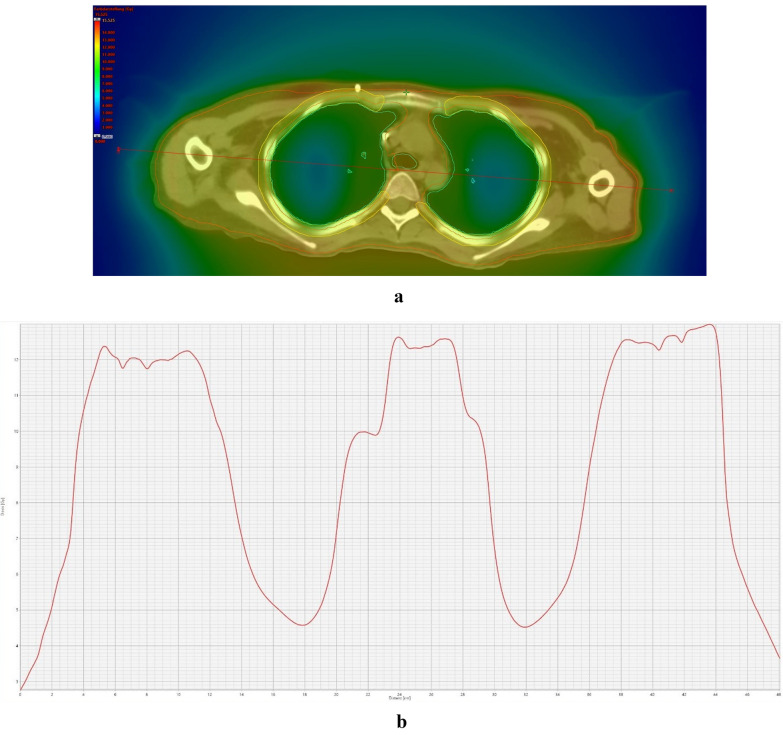
**a** Dose cross profile across entire PTV from right to left along the matching planning CT. **b** Dose cross profile across entire PTV from right to left in Gray and distance in cm

The difference between lung and ribcage doses are visualized in a DVH showing an example of the effect of lung sparing (Fig. 3a). By contrast, a simulation of a plan without lung sparing shows significant dose differences (Fig. 3b). In addition, a dose cross profile in coronal projection for an example TBI plan demonstrates the steep decrease in the dose received by the lungs relative to that received by the ribcage, mediastinum, and PTV (Fig. 4).

## Discussion

Due to its positive contribution to bone-marrow conditioning regimens, TBI is regularly used when preparing patients for allogenic BMT [17]. In combination with chemotherapy, TBI improves the long-term survival of patients with diseases, such as ALL or AML [18]. Given patients’ improved outcomes and lower relapse rate after TBI, it becomes increasingly important to reduce toxicity to OARs to prevent acute side-effects and long-term negative consequences. Lung tissue is particularly vulnerable to TBI, which can lead to interstitial pneumonitis, radiation pneumonitis, or pulmonary infection [13–15]. Thus, HT can be used to safely deliver radiation to patients undergoing BMT by reducing the dose received by lung tissue. Previous stimulation studies and studies using phantoms suggest that even with substantial dose sparing to lung tissue, all relevant structures in close proximity may be irradiated with the prescribed dose. Structures such as the ribcage are of particular importance, as they are one of the main targets of TBI or total marrow irradiation [7, 12].

Using data from actual patients treated at our institution, we examined the extent to which TBI delivered via HT reduces the dose of radiation received by lung tissue and the ribcage, as a substantial reduction in ribcage dose coverage could decrease the advantage of lung dose sparing. We found that the mean D_95_ for the lungs, indicating the minimum dose received by 95% of the lung volume, was reduced to 60.97% in the 4 Gy group, which was the smallest dose reduction observed across the three groups. This result could partially be explained by a lesser accumulation of tissue dose sparing due to only two treatment sessions but could also be an outcome of treatment planning. For patients in the 8 Gy and 12 Gy groups, mean D_95_ dropped to 54.77% and 37.44%, resulting in minimum doses of 4.38 Gy and 4.49 Gy, respectively. Shinde et al. propose a mean dose of < 8 Gy when delivering radiation to the lungs to reduce the possibility of pulmonary infection and radiation pneumonitis [14]. This outcome was achieved for all three groups in the present study, indicating that the risk of pulmonary toxicity was meaningfully reduced. However, the maximum D_5_ was close to the prescribed dose in all groups (101.63% for the 4 Gy group, 100.15% for the 8 Gy group, and 96.77% for the 12 Gy group), indicating a steep dose gradient. A complete vertical dose gradient is technically impossible, as some toxic effects, especially in higher dose areas, cannot be completely prevented. However, this is an expected result and does not reduce the feasibility of lung dose reduction.

To further support the viability of our approach, we also evaluated ribcage dose coverage. In the 4 Gy and 8 Gy groups, mean D_95_ was approximately 90%, which is still acceptably high, whereas mean D_50_ and D_5_ remained at the prescribed dose levels, ensuring good overall coverage of the ribcage. However, mean D_95_ was 78.65% in the 12 Gy group, which calls into question whether the relapse rate of these patients was elevated in comparison to that reported in the literature. It should be noted that although this variation is minor, it should be carefully considered in future analyses of patient relapse rates and toxic side effects.

In addition, mean HI was 1.17 for the 4 Gy group, 1.20 for the 8 Gy group, and 1.37 for the 12 Gy group. These values are well inside the range proposed by the Radiation Therapy Oncology Group, which recommends a maximum HI of  < 2 [12]. Therefore, even our maximum observed value of 1.76 in the 12 Gy group is still acceptable.

The need for dose reduction in patients receiving 4 Gy is questionable given that this dose may have minor toxic effects [19]. That is, a marginal reduction in lung tissue toxicity may not warrant the additional time needed to plan for lung sparing. Further studies are needed to determine toxicity levels and treatment success after lung sparing, although previous studies suggest positive outcomes [10].

This study addresses the challenge of sparing sensitive lung tissue while maintaining the prescribed dose to the ribcage during TBI. Although we demonstrate the technical effectiveness of this approach, further studies are needed to ensure that it does not increase the risk of disease recurrence or relapse. Additional research is also warranted to determine whether similar methods are feasible for planning and delineating other OARs, such as the liver, spleen, or kidneys. Furthermore, whereas 3D planning methods are already well-established for delivering TBI, our approach to reducing the dose to the lungs while maintaining the prescribed level to the ribcage could also be implemented when developing and establishing 4D planning approaches in the future.

## Conclusions

To improve the outcomes of patients undergoing TBI in preparation for allogeneic BMT, toxic effects on lung tissue can be reduced using HT to delineate, contour, and plan irradiation with lung dose sparing. The results of our study suggest that this approach does not compromise the quality of ribcage dose coverage, as dose homogeneity and overall coverage were consistent with guideline recommendations. These findings can inform clinicians’ planning and preparation for BMT to achieve optimal treatment outcomes.

## Data Availability

All data relevant to this publication have been included into the manuscript’s body.

## Abbreviations

ALL: Acute lymphoblastic leukaemia
AML: Acute myeloblastic leukaemia
BMT: Bone-marrow transplantation
CT: Computed tomography
HI: Homogeneity index
ICRU: International Commission on Radiation Units and Measurements
JT: Junctional target
PTV: Planning target volume
RTOG: Radiation Therapy Oncology Group
TBI: Total body irradiation
